# Supplementary material for: Plant metabolism of nematode pheromones mediates plant-nematode interactions
Source: Nat Commun. 2020 Jan 10;11:208. doi: 10.1038/s41467-019-14104-2 (PMC6954178; doi:10.1038/s41467-019-14104-2)
Supplement: Supplementary file 1 — Supplementary Information [file 41467_2019_14104_MOESM1_ESM.pdf]

Supplementary Figures and Figure Legends

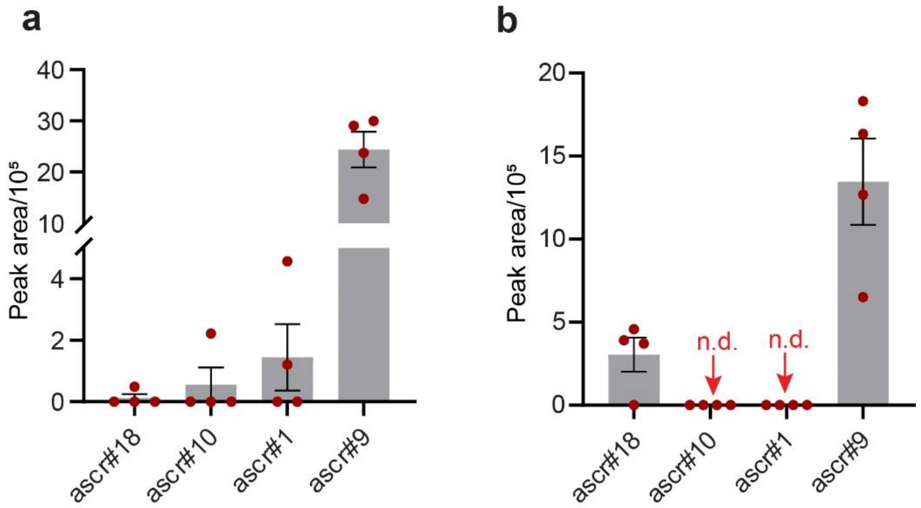

Supplementary Figure 1 | Accumulation and metabolism of ascr#18 in plants. (a-b) Accumulation of ascarosides in tomato **(a)** and wheat **(b)** roots treated with 10 and 50 nM ascr#18 for 24 h, respectively. Abundances of ascarosides are shown as the peak area, as measured in LC-MS. Data are averages ± SEM (n = 4). (n.d. = not detected). Source data are provided as a source data file.

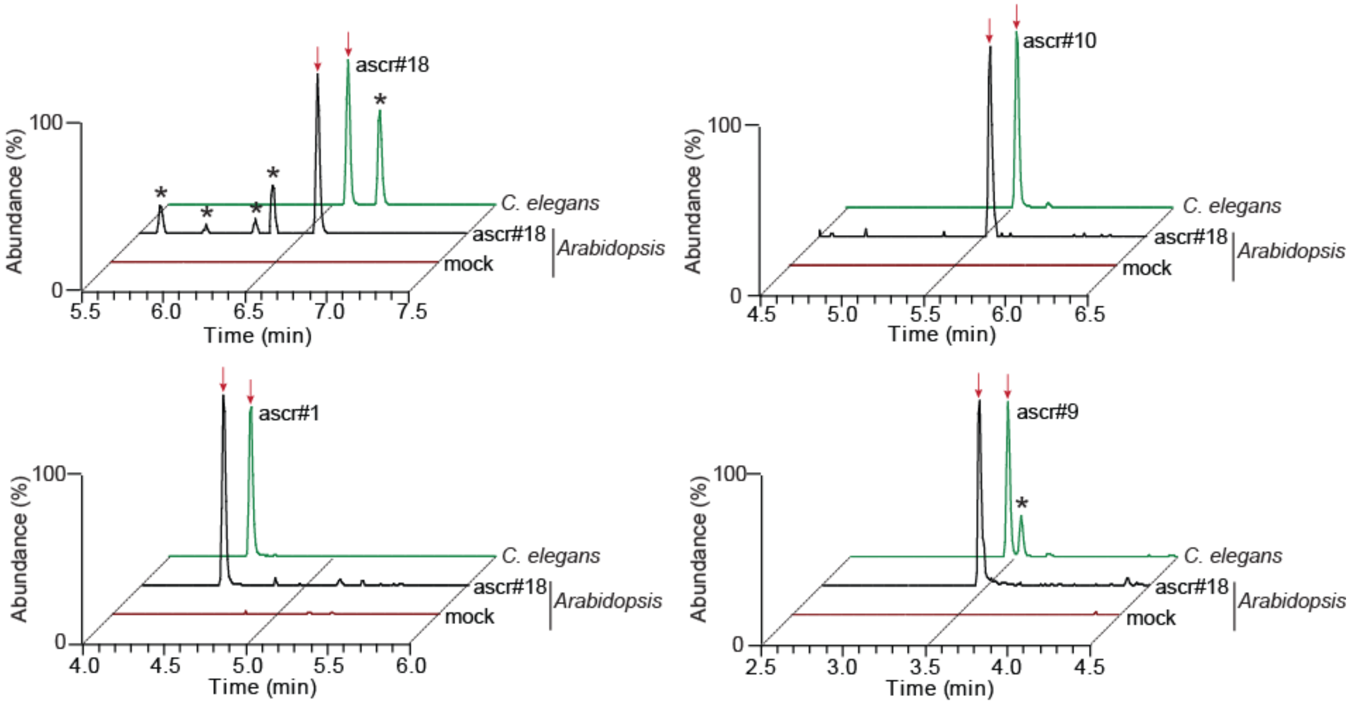

Supplementary Figure 2 | Confirmation of ascarosides identity in plants. LC-MS analysis of exudates from tomato roots mock treated or treated with 1 µM ascr#18 for 24 h and from *Caenorhabditis elegans*, showing extracted ion chromatograms [EIC] in ESI$^-$ of ascr#18, ascr#10, ascr#1, and ascr#9. Peaks marked with an asterisk represent unrelated metabolites of similar m/z.

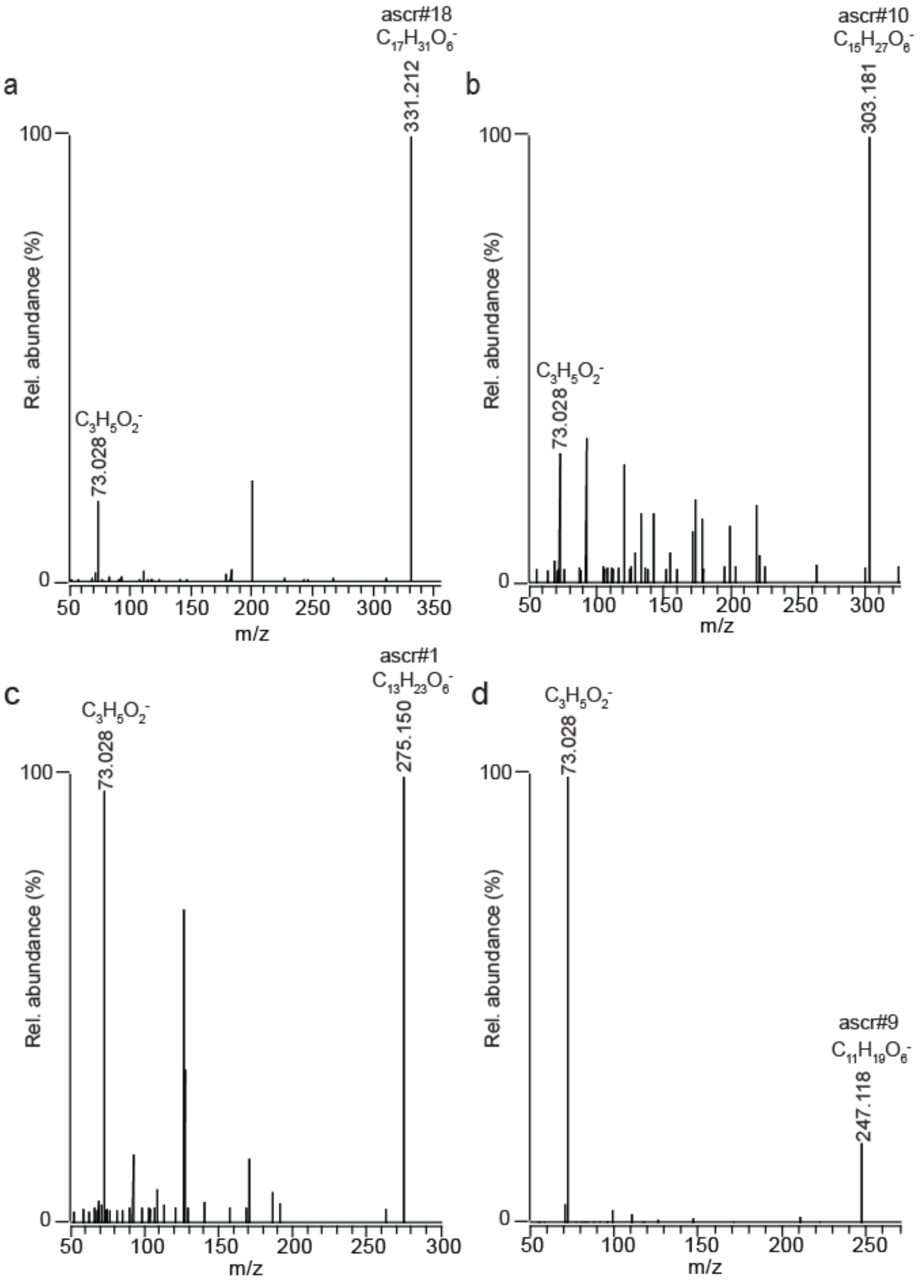

Supplementary Figure 3 | LC-MS/MS analyses of *Arabidopsis* roots. LC-MS/MS spectra of ascr#18 (**a**), ascr#10 (**b**), ascr#1 (**c**), and ascr#9 (**d**) in ESI^- showing presence of diagnostic product ion at m/z = 73.028 $[C_3H_5O_2]^-$, which originates from the ascarylose unit of ascarosides. The method has been previously used for the detection of ascarosides in *C. elegans*[1].

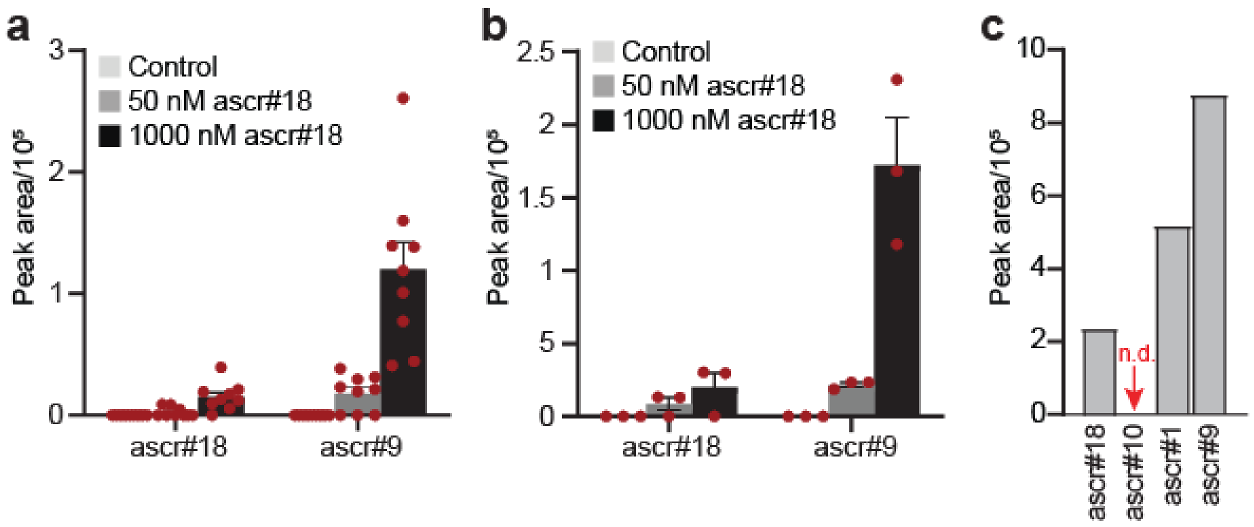

Supplementary Figure 4 | Metabolism of naturally acquired ascr#18 by plants. (a-b) LC-MS analysis of *Arabidopsis* **(a)** and tomato **(b)** roots from plants grown in soil and treated with 50 nM and 1 µM ascr#18 or treated with mock solution for 48 h. Abundances of ascarosides are shown as the peak area, as measured in LC-MS. Data are mean ± SEM (n = 9 for *Arabidopsis* and n=3 for tomato). **(c)** LC-MS analysis of exudate of tomato plant roots naturally infested with plant parasitic nematodes (*M. incognita*). Abundances of ascarosides are shown as peak area, as measured in LC-MS.

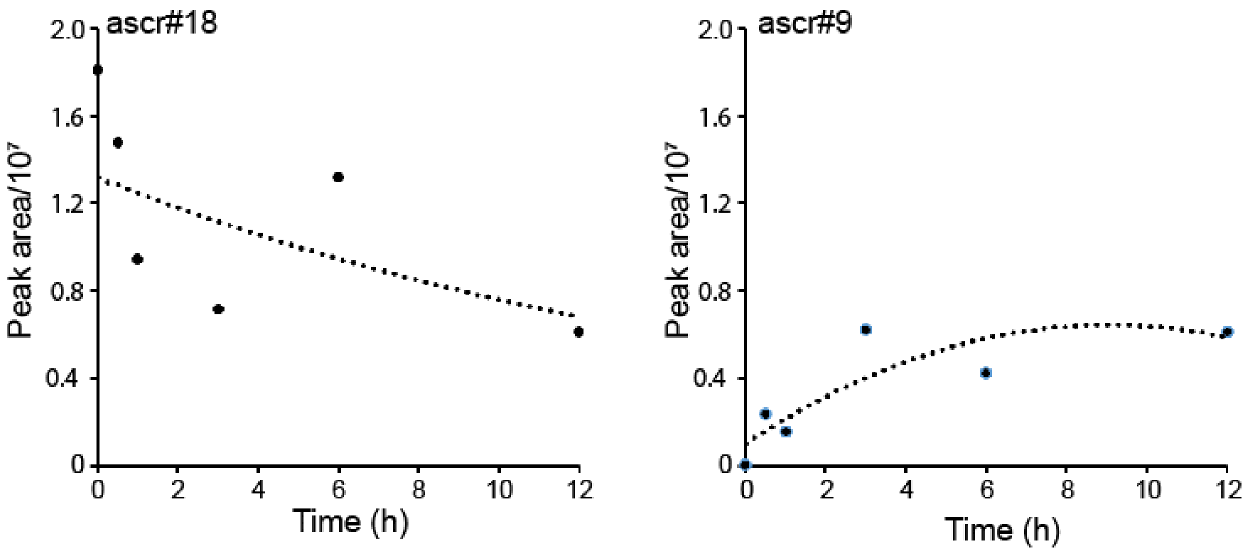

Supplementary Figure 5 | LC-MS analyses of tomato leaves. LC-MS analysis of tissue of tomato leaves infiltrated with 1 µM ascr#18; tissue was collected over a 12 h period. Abundances of ascr#18 and ascr#9 are shown as the peak area, as measured in LC-MS. Source data are provided as a source data file.

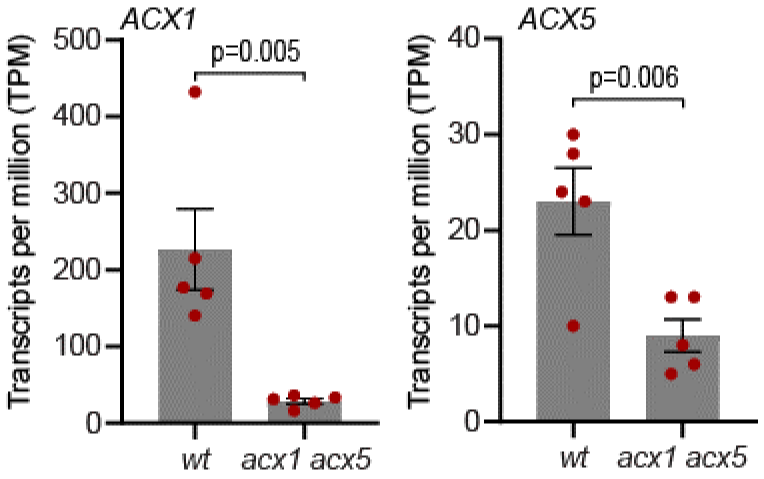

Supplementary Figure 6 | Transcript level of *ACX1* and *ACX5*. RNAseq analysis of ten day-old *Arabidopsis* wildtype and *acx1 acx5* roots. TNA-Seq counts was normalized and plotted as Transcripts per million (TPM). Data are mean ± SEM (n = 5). two-tailed *t*-test. Source data are provided as a source data file.

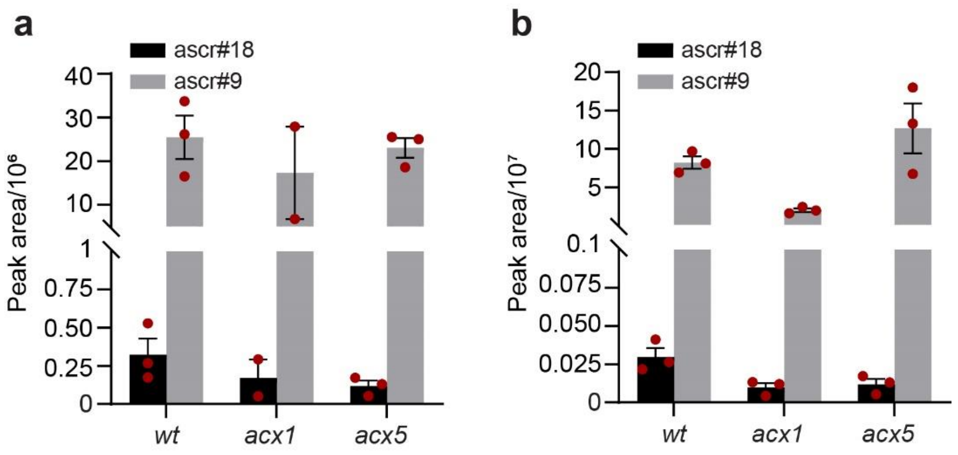

Supplementary Figure 7 | The abundance of ascarosides in *wt, acx1, and acx5 Arabidopsis* plants. (a-b) LC-MS analysis of *wt*, *acx1*, and *acx5 Arabidopsis* roots treated with 250 nM (**a**) and 1 µM (**b**) of ascr#18 for 24 h. Abundances of ascarosides are shown as the peak area, as measured in LC-MS. Data are mean ± SEM (n = 3)

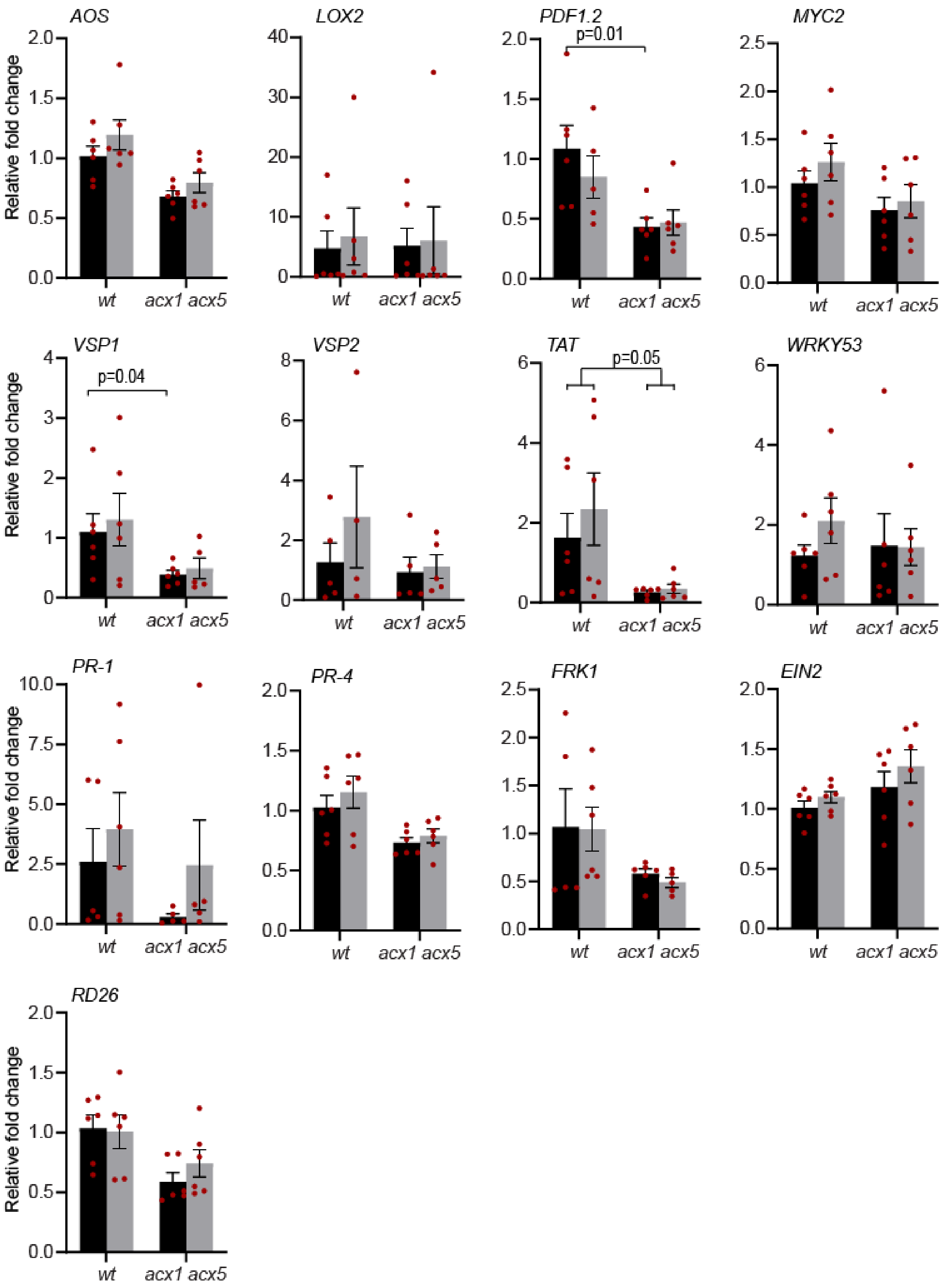
AOS
LOX2
PDF1.2
MYC2
VSP1
VSP2
TAT
WRKY53
PR-1
PR-4
FRK1
EIN2
RD26
Relative fold change
p=0.01
p=0.04
p=0.05
wt
acx1 acx5

Supplementary Figure 8 | At a concentration of 50 nM, ascr#18 does not activate defense responses in *Arabidopsis* roots. Expression of defense-related genes in *Arabidopsis* roots 48 h after treatment with 50 nM ascr#18. Transcript level of jasmonic acid signaling genes (*PDF1.2*, *AOS*, *LOX2*, *MYC2* (*Jasmonate insensitive 1*), *Vegetative storage protein 1, 2* (*VSP1*, *VSP2*), and *Tyrosine Aminotransferase* (*TAT*)), salicylic acid signaling genes (*PR-1* and *PR-4*), MAPK signaling related *FRK1*, transcription factor *WRKY53* (involved in both JA and SA signaling), ethylene signaling gene *Ethylene sensitive 2* (*EIN2*) and absicic acid signaling gene *Responsive to Dessication 26* (*RD26*) were determined by qRT-PCR. Data are mean±SEM ($n \geq 4$). two-tailed *t*-test. Source data are provided as a source data file.

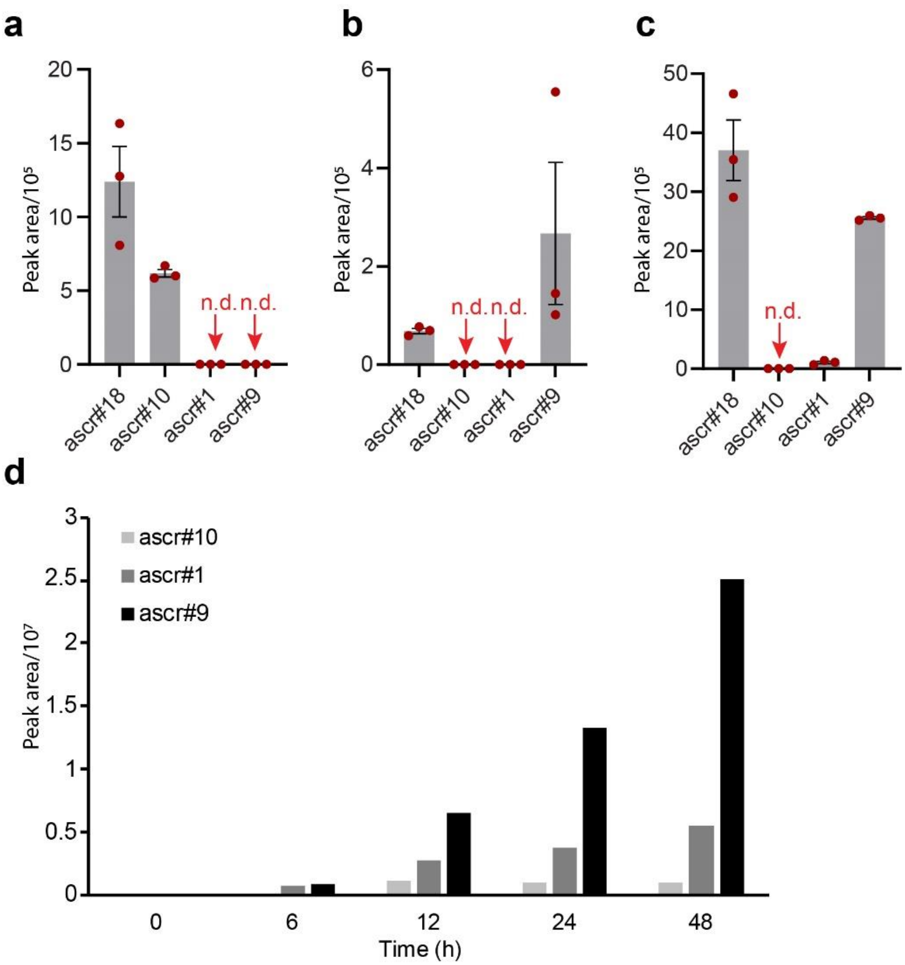

Supplementary Figure 9 | The abundance of ascarosides in root exudates. (a) Relative abundances of ascarosides in root exudates of ten-day old *Arabidopsis acx1 acx5* mutant seedlings treated with 10 nM ascr#18 for 6 h. Exudates from approximately 40 seedlings were collected in distilled water for 1 h and pooled in one tube. Abundances of ascarosides are shown as the peak area, as measured in LC-MS. Data are average ± SEM (n = 3), n.d. = not detected. **(b-c)** Relative abundances of ascarosides in root exudates of eight-day old tomato seedlings treated with 10 nM (**b**) or 1000 nM (**c**) ascr#18 for 6 h. Exudates from approximately 10 seedlings were collected in distilled water for 1 h and pooled in one tube. Abundances of ascarosides are shown as the peak area, as measured in LC-MS. Data are average ± SEM (n = 3), n.d. = not detected. **(d)** Relative abundances of ascr#10, ascr#1, and ascr#9 in the growth media of eight-day old tomato seedlings supplemented with 1 µM ascr#18. Ten-gram media was collected over a 48 h period for metabolome extraction. Abundances of ascarosides are shown as the peak area, as measured in LC-MS. Source data are provided as a source data file.

Supplementary Table 1: Primers used in this study.

Primer Name	Sequence
AtPR-1 F	TCGTCTTTGTAGCTCTTGTAGGTG
AtPR-1 R	TAGATTCTCGTAATCTCAGCTCT
AtPDF1.2-F	TCATGGCTAAGTTTGCTTCC
AtPDF1.2-R	AATACACACGATTAGCACC
AtFRK1-fw	TGCAGCGCAAGGACTAGAG
AtFRK1-rv	ATCTTCGCTTGGAGCTTCTC
AtPHI-fw	TTGGTTTAGACGGGATGGTG
AtPHI-rv	ACTCCAGTACAAGCCGATCC
AtUBQ10-fw	GGCCTTGTATAATCCCTGATGAATAAG
AtUBQ10-rv	AAAGAGATAACAGGAACGGAAACATAG
AtPR4-F	CTGGACCGCCTTCTGCGGG
AtPR4-R	AGCCTCCGTTGCTGCATTGGT
AtAOS-F	TCTTCTCTTCGCCACGTGC
AtAOS-R	GGTTATGAACTTGATGACCCGC
AtLOX2-F	TTGCTCGCCAGACACTTGC
AtLOX2-R	GGGATCACCATAAACGGCC
AtVSP1-F	ACTGGTCGTGGTTAGAGTCC
ATVSP1-R	CTCCAATATTCCCAACGATG
AtVSP2-F	TCCATCAACTACGCCAACT
AtVSP2-R	GGAGGGTATCATCTAGTACAA
AtMYC2-F	CTGGCAACCGTCGTATGATTTCT
AtMYC2-R	AACCATTCCGTATCCGTCACCTC
AtTAT-F	TGGCTCTAGGGGCAGAGAAT
AtTAT-R	CCTTGGAGATGGCATGACGA
AtWRKY53-F	TCACTTTTTCTGACCACTTTGG
AtWRKY53-R	AAGGAAGAGATATGTTAAGTTGGG
AtEIN2-F	GGTGTGCGTCTTATGGTCG
AtEIN2-R	AACTGCTCAAAGGGCTGTCTGG
AtRD26-F	TTATTGGAAAGCAACGGGTA
AtRD26-R	TCGTCAAGCTGTGATGAAGA
AtERF1-F	CTAATCGAGCAGTCCACGCAACA
AtERF1-R	GTCCCGAGCCAAACCCTAATACC
AtMPK3-F	ATGCGCTTATTGACAGAGTT
AtMPK3-R	TCAGAGCTTGTTCAACAGTG
